# Modified oxymatrine as novel therapeutic inhibitors against Monkeypox and Marburg virus through computational drug design approaches

**DOI:** 10.1111/jcmm.70116

**Published:** 2024-09-28

**Authors:** Md. Rezaul Islam, Suvro Biswas, Ummy Amena, Miadur Rahman, Shirmin Islam, Md. Ariful Islam, Md. Abu Saleh, Hesham M. Hassan, Ahmed Al‐Emam, Magdi E. A. Zaki

**Affiliations:** ^1^ Department of Pharmacy, Faculty of Allied Health Sciences Daffodil International University Ashulia Dhaka Bangladesh; ^2^ Department of Genetic Engineering and Biotechnology University of Rajshahi Rajshahi Bangladesh; ^3^ Department of Pharmacy, Faculty of Life & Earth Sciences Jagannath University Dhaka Bangladesh; ^4^ Department of Pharmaceutical Sciences North South University Dhaka Bangladesh; ^5^ Department of Pathology, College of Medicine King Khalid University Asir Saudi Arabia; ^6^ Department of Chemistry, College of Science Imam Mohammad Ibn Saud Islamic University Riyadh Riyadh Saudi Arabia

**Keywords:** ADMET, DFT, Marburg virus, molecular docking, molecular dynamics simulation, Monkeypox

## Abstract

Global impact of viral diseases specially Monkeypox (mpox) and Marburg virus, emphasizing the urgent need for effective drug interventions. Oxymatrine is an alkaloid which has been selected and modified using various functional groups to enhance its efficacy. The modifications were evaluated using various computatioanal analysis such as pass prediction, molecular docking, ADMET, and molecular dynamic simulation. Mpox and Marburg virus were chosen as target diseases based on their maximum pass prediction spectrum against viral disease. After that, molecular docking, dynamic simulation, DFT, calculation and ADMET prediction were determined. The main objective of this study was to enhance the efficacy of oxymatrine derivatives through functional group modifications and computational analyses to develop effective drug candidates against mpox and Marburg viruses. The calculated binding affinities indicated strong interactions against both mpox virus and Marburg virus. After that, the molecular dynamic simulation was conducted at 100 ns, which confirmed the stability of the binding interactions between the modified oxymatrine derivatives and target proteins. Then, the modified oxymatrine derivatives conducted theoretical ADMET profiling, which demonstrated their potential for effective drug development. Moreover, HOMO‐LUMO calculation was performed to understand the chemical reactivity and physicochemical properties of compounds. This computational analysis indicated that modified oxymatrine derivatives for the treatment of mpox and Marburg virus suggested effective drug candidates based on their binding affinity, drug‐like properties, stability and chemical reactivity. However, further experimental validation is necessary to confirm their clinical value and efficacy as therapeutic candidates.

## 1 INTRODUCTION

The Monkeypox virus (mpox), classified as a zoonotic DNA virus and belonging to the *Poxviridae* family and *Orthopoxvirus* genus, causes a potential disease in humans with similarities to smallpox and a fatality rate of approximately 10%. The first case of mpox in humans was identified in the Democratic Republic of the Congo in 1970.^1^, ^2^ Subsequently, more human cases had also been reported in the Republic of Congo and Sudan, indicating a potential expansion of the virus's natural global distribution.^3^ Notably, the first recorded case of mpox involved a 9‐month‐old newborn, highlighting the susceptibility of young children to this infection. In fact, between 1971 and 1997, approximately 83% of mpox cases in the Democratic Republic of the Congo occurred in children under the age of five.^4^, ^5^ The World Health Organization (WHO) has received reports from 50 countries and territories detailing 3413 laboratory‐confirmed illnesses and one fatality between January 1 and June 22, 2022.^6^ Besides, another recent report has announced by WHO that a total number of 12,569 infected patient were confirmed in 156 health zones in 22 out of 26 (85%) provinces in the Democratic Republic of the Congo between 1 January and 12 November 2023, where more than 581 patients were died [ratio: 4.6%].^7^

Second, another highly virulent disease that causes hemorrhagic fever are infected in global people known as Marburg virus disease (MVD). and its fatality rate can reach up to 90% among those infected.^8^, ^9^ In August 2021, an outbreak of MVD was reported in the Republic of Guinea. This was the first‐ever case of the Marburg virus reported in Guinea and West Africa.^10^, ^11^

Equatorial Guinea reported a recent outbrea of suspected and laboratory‐confirmed MVD cases between February 13 and May 1, 2023. During this period, there were 23 suspected cases, 17 laboratory‐confirmed cases, and 12 fatalities were reported. Similarly, between March 16 and April 30, 2023, the United Republic of Tanzania recorded 9 cases, including 8 laboratory‐confirmed cases and 1 suspected case, with the last verified case reported on April 11.^12^

Although these two viral infections are emerging as potential epidemic threats, there are currently no approved treatments available for them. To mitigate the impact and prevent the spread of these new pandemics, urgently need an effective therapeutic agent, or new drugs.^13^, ^14^, ^15^

Oxymatrine is a quinazoline alkaloid derived from the *Sophora flavescent* plant, which demonstrates various therapeutic properties such as organ protection, anti‐cancer activity, anti‐inflammation, and anti‐viral effects against HCV and HBV. Oxymatrine has also demonstrated inhibitory activity against the Influenza virus and Coxsackievirus.^16^, ^17^, ^18^, ^19^ Therefore, Oxymatrine has been selected for analog design by changing the functional groups, to identify a new compounds and assess its effectiveness against mpox and Marburg virus by employing computational approaches. Computational approaches and methods used the evaluation of pharmacokinetic properties that could be helpful in the initial stages of anti‐viral drug development.^20^, ^21^ This computational pipeline aims to repurpose novel drugs against the Marburg and mpox viruses.

## 2 MATERIALS AND METHODS

### 2.1 Pass prediction

Pa (probability of activity) and Pi (probability of inactivity), which stand for the primary screening of novel drug development, are used in the pass prediction. The chemical structure was entered into the pass online website to collect the pass prediction data using the link “http://way2drug.com/PassOnline/predict.php”^22^. We measured anti‐viral, antifungal, antibacterial and antineoplastic properties during our work.

### 2.2 Determination of the data of ADMET

The PkCSM is a website that has been utilized to predict and collect the pharmacokinetic and toxicological data for small‐molecule medicines through the chemical structure. PkCSM functions as a platform that uses to analyse the numerous elements of *in silco* drug development. Researchers and scientists can access essential data on the pharmacokinetic behaviour and possible toxicity of small‐molecule drugs by using this website. It provides to analyse and understand chemical structures,^23^ which enables the prediction of critical features that influence the effectiveness and safety of pharmaceuticals which can be accessed at http://biosig.unimelb.edu.au/pkcsm/. It also offers a vast array of biological and pharmacological data, including information on water solubility, Caco‐2 permeability, human intestinal absorption, the volume of distribution (VDss), BBB permeability, CYP450 1A2 inhibitors, CYP450 2C skin sensitization, Hepatotoxicity, Renal OCT2 substrate AMES Toxicity, etc.^24^

### 2.3 Structure activity relationship and molecular optimization

**FIGURE 1:**
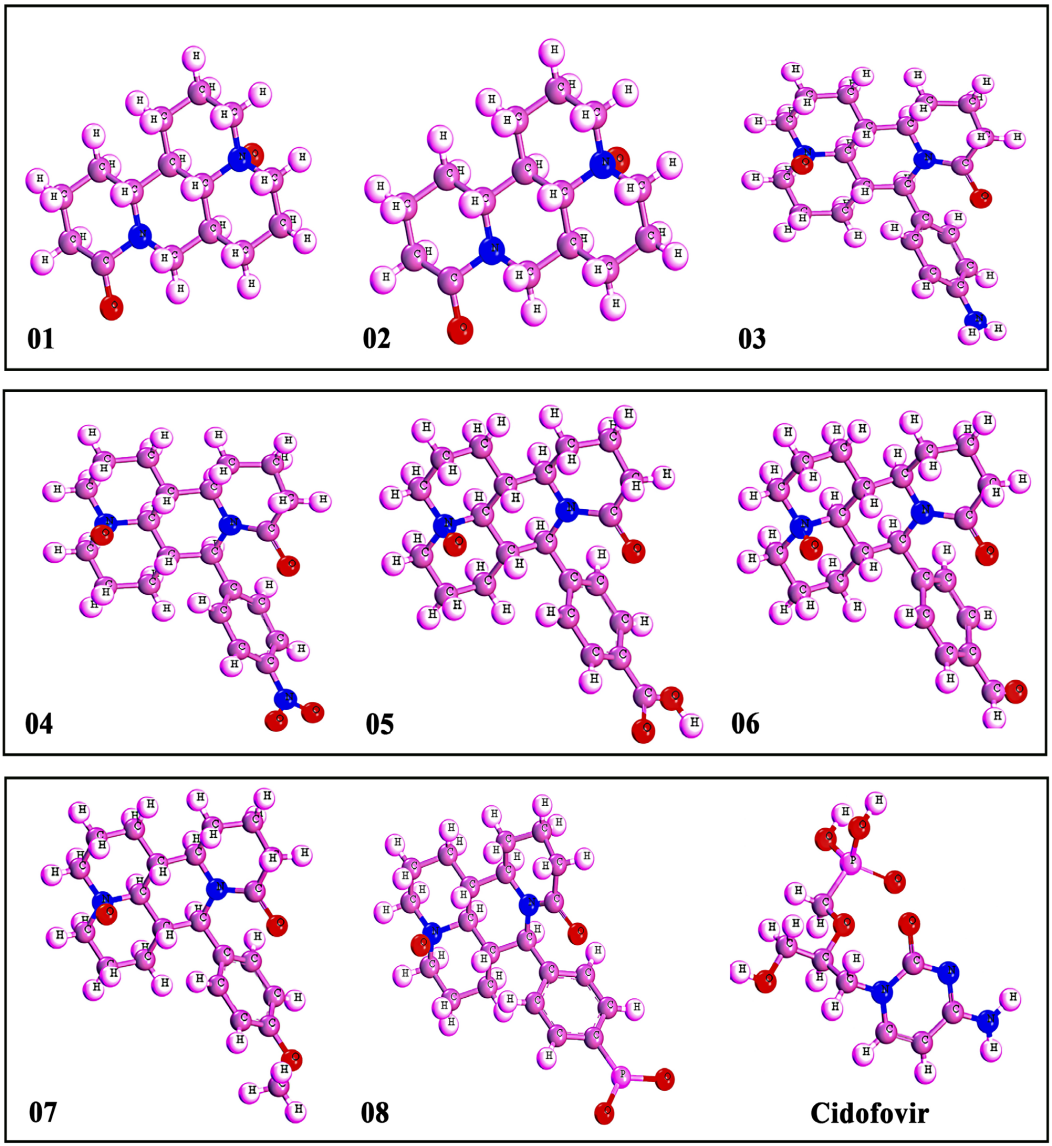
Three‐dimensional structure of oxymatrine and its derivatives.

Structure–Activity Relationship, often known as SAR, is a technique that seeks to improve the efficiency of the ligand by modification of their structures. Essential stages in the search for therapeutic possibilities or new drug candidates include the synthesis of ligands/ designing new molecules by SAR. So, the oxymatrine has been modified by adding several functional groups, including benzene, NH_2_, NO_2_, COOH, CHO, OCH_3_ and PO_3_ (Figure 1). Then, the chemical descriptor, quantum properties, and geometrical optimization were determined using the DFT functional of the DMol3 code with a DNP basis set.^25^ Following the optimization process, the optimized molecules were saved for further analysis, and HOMO‐LUMO Various properties were then calculated. These properties include the frontier molecular orbital energies (ϵLUMO and ϵHOMO), the energy gap (ΔE), hardness (η), and softness (s).

### 2.4 Lipinski rule, pharmacokinetics and drug‐likeness

The Lipinski rule, pharmacokinetics, and drug‐likeness are essential factors in the development of new drugs, especially for oral medications. Therefore, the Lipinski rule was evaluated using the SwissADME online tool at “http://www.swissadme.ch/index.php.” In terms of Lipinski rule, the most important measurements were those of Molecular weight, the number of Hydrogen bond acceptor and donor, and Octanol–water partition coefficient etc. are analysed.^26^, ^27^

### 2.5 Protein preparation

**FIGURE 2:**
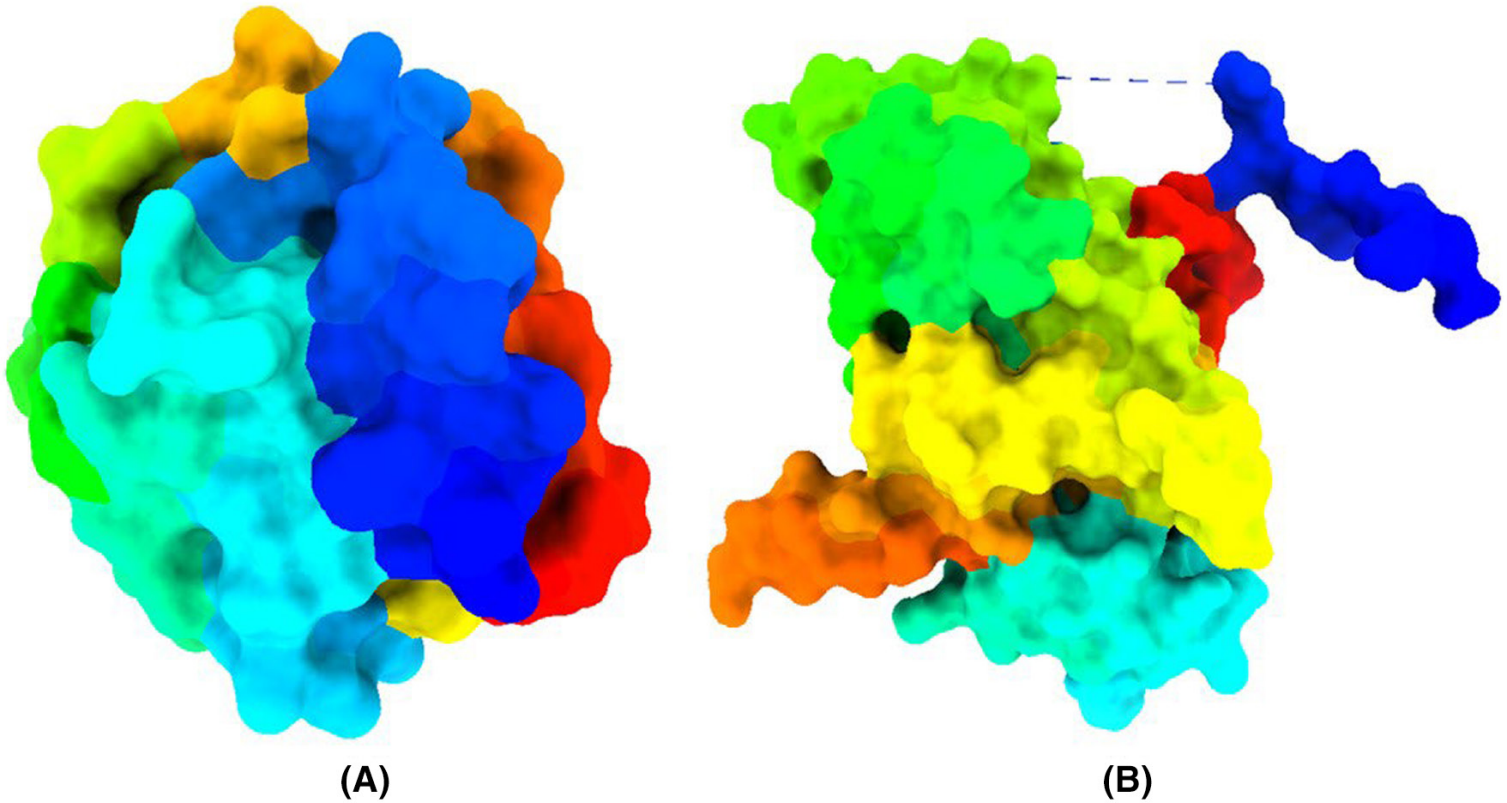
Three‐dimensional protein structure of Monkeypox (A) and Marburg virus (B).

Two pathogenic targeted proteins structures were downloaded from the Protein Data Bank (PDB) [https://www.rcsb.org/], A42R Profilin‐like Protein from mpox Virus Zaire‐96‐I‐16 (PDB ID 4QWO) and Marburg virus VP24 (PDB 4OR8).^28^, ^29^ The structure of A42R closely resembles, which are proteins found in cells known to play a role in regulating the formation of actin cytoskeletons. VP24 is a nucleocapsid‐associated protein that may inhibit interferon gene suppression and counteract the anti‐viral functions mediated by interferon, particularly by blocking the nuclear import of signal transducer and activator of transcription 1 (STAT1).^29^, ^30^, ^31^, ^32^ These two biological macromolecules were selected as potential targets for mpox and Marburg Virus (Figure 2). Upon downloading the protein structures, they were imported into biovia discovery studio and Pymol application, and prepared for the molecular docking. This protein preparation process involved the removal of excess water and heteroatoms to obtain a clean protein structure. These elements were removed to reduce computational complexity and provide a clearer view of the binding site. Furthermore, undesirable heteroatoms that may have been attached to the receptor could potentially occupy the active site.

### 2.6 Molecular docking method

Molecular docking was primarily employed to investigate the binding affinity of selected ligands with the targeted receptor. For the docking process, AutoDock Vina was integrated with PyRx Virtual Screening Tools.^33^ Regarding dimensions (in Å), the grid center points were set at *X* = −33.4882, *Y* = −21.6558 and *Z* = 49.8741, while the grid size was adjusted (in Å) to *X* = 49.2566, *Y* = 43.68, *Z* = 40.54. The dimensions of the grid box were selected and fine‐tuned to incorporate the substrate‐binding region of the protein. The non‐covalent interactions between the pathogenic protein and the ligands were visualized using the BIOVIA Discovery Studio Visualizer.^34^

### 2.7 Molecular dynamics simulation

YASARA software through the AMBER14 force field assistance was used for the molecular dynamics (MD) simulation.^35^, ^36^, ^37^ Primitively, the docked complexes were subjected to optimization. Here, the TIP3P solvation model was employed because of the construction of the cubic simulation utilizing a periodic boundary state.^38^, ^39^ In addition, approximately 20‐Å extension of the simulation cell in both paths was accomplished from the docked complexes of protein and ligand. It is noted that the physiological condition regarding the simulation cell was set as pH 7.4, 298 K, and 0.9% NaCl. Approximately 5000 cycles concerning the steepest gradient algorithm were employed to accomplish the primal energy minimization by dint of the simulated annealing strategy.^40^, ^41^ However, the time step regarding the simulation cell was fixed as 1.25 fs. A cut‐off radius (8.0 Å) in tandem with the Particle Mesh Ewald approach was utilized to enumerate the long‐range associated electrostatic interactions.^42^, ^43^, ^44^ After every 100 ps, the simulation trajectories were reserved. A consistent temperature and pressure in conjunction with the Berendsen thermostat were operated to execute the simulation for 100 ns.^45^, ^46^ Eventually, the RMSD or root mean square deviation, Rg or radius of gyration, hydrogen bond, SASA or solvent accessible surface area, and RMSF or root mean square fluctuation was analysed by the utilization of trajectory data.^47^, ^48^, ^49^ Moreover, MM‐PBSA (Molecular Mechanics‐Poisson Boltzmann Surface Area) approaches were employed to calculate the binding energy from the simulation snapshots by using the following equation.^50^

$$\text{Binding Energy}=E_{\text{potRecept}}+E_{\text{solvRecept}}+E_{\text{potLigand}}+E_{\text{solvLigand}}-E_{\text{potComplex}}-E_{\text{solvComplex}}$$

MM‐PBSA binding energy calculation was carried out using the YASARA macro, where a higher positive energy indicates a better binding.^51^

Additionally, the binding free energy has been computed in the YASARA dynamics software package using the MM‐PBSA approach and the AMBER14 force field.^52^, ^53^ Using the following equation, the default macro file was modified to calculate the binding free energy:

$$\text{∆Gbind}={\text{∆Gcomplex}}_{\left(\text{minimised}\right)}–[{\text{∆Gligand}}_{\left(\text{minimised}\right)}+{\text{∆Greceptor}}_{\left(\text{minimised}\right)]}$$

$$\text{∆Gbind}={\text{∆G}}_{\mathrm{MM}}+{\text{∆G}}_{\mathrm{PB}}+{\text{∆G}}_{\mathrm{SA}}-T∆S$$

where, the sum of electrostatic and van der Waals interactions are indicated by ∆G_MM_; the entropic contribution is indicated by T∆S; the non‐polar and polar solvation energies are indicated by ∆G_SA_ and ∆G_PB_, respectively.^50^

## 3 RESULTS AND DISCUSSION

### 3.1 Pass prediction spectra analysis

When designing new molecules through computational or in silico approaches, it is often unclear which disease they will effectively combat. In such cases, Pass prediction analysis helps us identify a specific biological activity.^51^

A drug with a high Pa score against a specific biological activity suggests that this molecule might be capable to inhibit that specific disease. Based on the Pass prediction data for reported compounds, the Pa scores for antiviral (Hepatitis B), most of them are above 0.800 where the antibacterial agents fall within the range of 0.178 to 0.366, and none of the compounds were active against antifungal, respectively (Table 1). Finally, antineoplastic agents are notably ineffective. Consequently, the medication may prove beneficial in combating viral pathogens, as indicated by the presented findings, and selected the two viral target proteins as a receptor.

**TABLE 1:** PASS calculated data of oxymatrine and its derivatives.

No	Antiviral (Hepatitis B)		Antifungal		Antibacterial		Antineoplastic	
	Pa	Pi	Pa	Pi	Pa	Pi	Pa	Pi
L01	0.430	0.011	–	–	0.178	0.138	0.327	0.137
L02	0.907	0.001	–	–	0.253	0.081	0.594	0.046
L03	0.888	0.001	–	–	0.314	0.055	0.589	0.048
L04	0.888	0.001	–	–	0.284	0.066	0.539	0.060
L05	0.877	0.001	–	–	0.366	0.039	0.454	0.086
L06	0.885	0.001	–	–	0.337	0.047	0.650	0.035
L07	0.879	0.001	–	–	0.226	0.097	0.593	0.047
L08	0.906	0.001	–	–	–	–	0.566	0.053

### 3.2 Lipinski rule and drug‐likeness

Drug‐likeness, which determines whether a given compound qualifies as a drug or a non‐drug like molecules. The activity of a compound in a physiological system is influenced by several factors, including lipophilicity, electronic dispersion, hydrogen bonding traits, molecule size and flexibility, and the presence of different pharmacophore aspects. The Lipinski rule is employed to address these presumptions of drug‐likeness. The significance of the Lipinski Rule lies in its ability to quickly determine whether a compound is likely to possess favourable pharmacokinetic properties or not. This technique plays essential rule in prioritizing and selecting promising drug candidates that have a higher probability of success in subsequent stages of drug development, saving time and resources during clinical and pre‐clinical trials.^54^, ^55^, ^56^, ^57^

According to the Lipinski rule, pharmacokinetics, and drug‐likeness: the molecular weight must be less than 500 g/mol, the calculated octanol/water partition coefficient must be less than 5 (LogP), the number of hydrogen bond donors must be just under 5, and the number of hydrogen bond acceptors (particularly N and O atoms) must not exceed 10. The molecules ought to have taken oral medication into account if they followed this rule. It has been determined that the reported molecules in our current research with a molecular weight between 264.36 and 402.42 Dalton, hydrogen bond acceptor or donor between 0 and 4 and Octanol–water partition coefficient (Clog P) ranges are 0.46 to 1.56 excluding ligand 08 and based on this data, the Lipinski rule had satisfied by all molecules. They should therefore be viewed as promising compounds for oral medications (Table 2).

**TABLE 2:** Data of lipinski rule, pharmacokinetics and drug likeness properties of the selected ligands.

Ligand No	Molecular weight	Hydrogen bond acceptor	Hydrogen bond donor	Octanol–water partition coefficient (Clog P)	Lipinski rule	
					Result	Violation
01	264.36	02	00	0.46	Yes	00
02	340.46	02	00	1.56	Yes	00
03	355.47	02	01	1.02	Yes	00
04	385.46	04	00	1.38	Yes	00
05	384.47	04	01	1.15	Yes	00
06	368.47	03	00	1.29	Yes	00
07	370.49	03	00	1.56	Yes	00
08	402.42	04	00	−4.10	Yes	00

### 3.3 Molecular docking analysis against mpox and Marburg virus

The use of molecular docking studies is very important to identify the interactions which has been formed between prospective therapeutic molecules and target receptor. Simply described, docking is a computational modelling technique used to predict how a protein or medication might interact and formed a complex.^58^, ^59^ In this investigation, mpox and the Marburg virus, have been selected as target receptor, and investigated the possible binding affinity by using molecular docking analysis. Our designed compounds exhibit binding energies ranging from −7.1 to −8.1 kcal/mol against the mpox virus (PDB ID 4QWO) and from −6.8 kcal/mol to −7.2 kcal/mol against the Marburg virus (PDB 4OR8) (Table 3). In contrast to the standard and modified structures of oxymatrine derivatives, the standard (cidofovir) offered −6.0 kcal/mol and − 6.1 kcal/mol, respectively.

**TABLE 3:** Binding Affinity of the selected ligands with a standard against Monkeypox and Marburg virus proteins.

No	Monkeypox Virus (PDB ID 4QWO)	Marburg virus (PDB 4OR8)
	Binding affinity (kcal/mol)	Binding affinity (kcal/mol)
01	−7.1	−7.2
02	−7.3	−7.0
03	−7.4	−6.8
04	−7.7	−7.3
05	−8.0	−7.1
06	−7.5	−6.9
07	−7.6	−7.2
08	−8.1	−7.0
Standard (Cidofovir)	−6.0	−6.1

A significantly improved outcome was observed with oxymatrine and its derivatives, indicating that these molecules may have inhibited the replication of the pathogenic Marburg and mpox viruses. Following this molecular docking analysis, top two compounds were selected for molecular dynamic simulations (complexes 05 and 08 against the mpox virus), and (complexes 04 and 07 against the Marburg virus), to assess their stability. Our focus extended to both binding affinity and the identification of active amino acid residues involved in the formation of drug‐protein complexes.

After molecular docking, we utilized Discovery Studio to visualize the active sites. Notably, the ligand‐protein complex 01 comprised only six active amino acid residues, whereas the ligand‐protein complex 07 comprised a total of eight active amino acid residues, surpassing the number in ligand 01. A far better outcome was obtained with oxymatrine and its derivatives, suggesting that these molecules may have prevented the pathogenic Marburg and mpox viruses from reproducing.

### 3.4 Protein‐ligand interaction analysis

**FIGURE 3:**
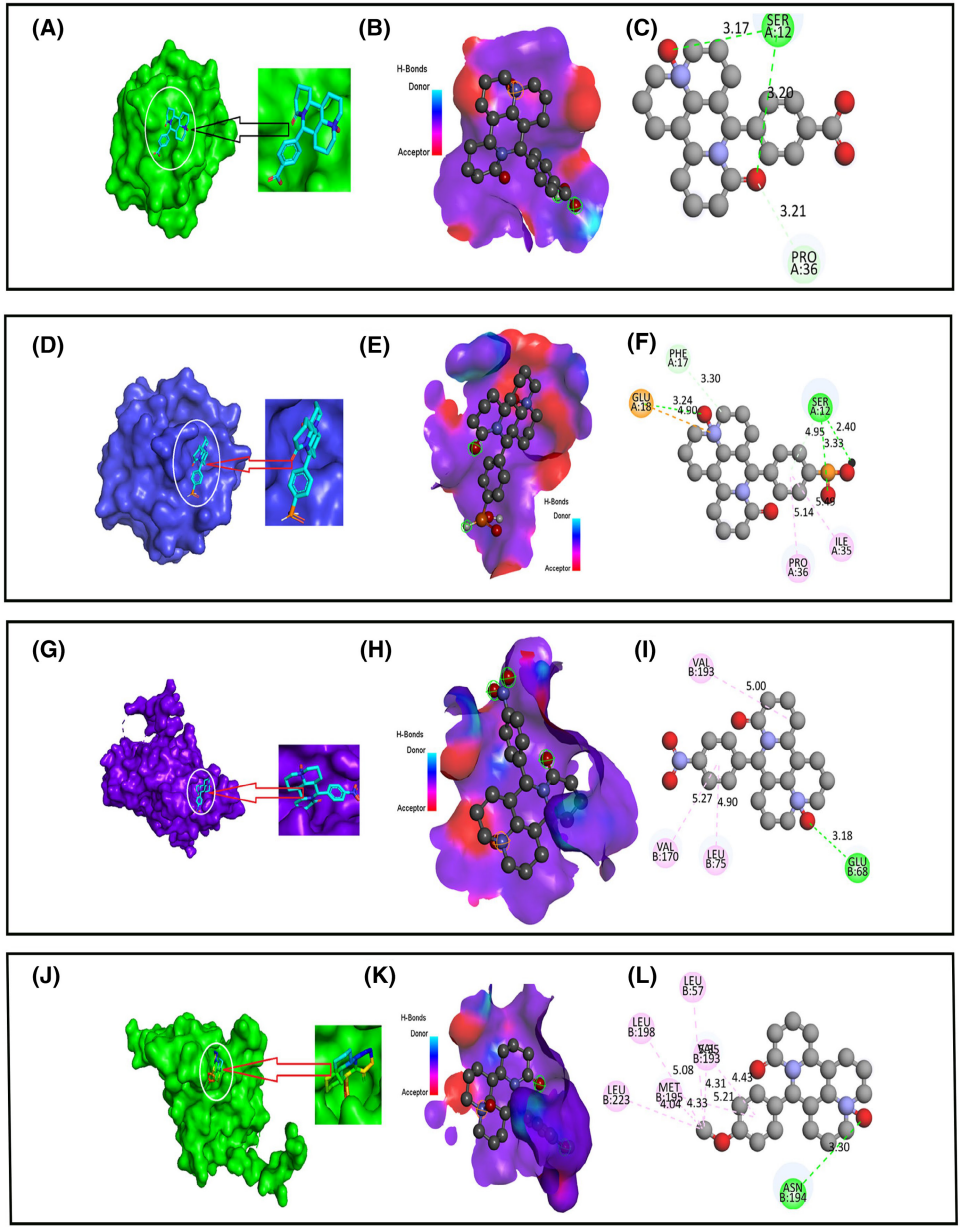
Molecular docking poses of Monkeypox Virus (PDB ID 4QWO) with Ligand 05 and Ligand 08 (A–F) and Marburg virus (PDB 4OR8) with Ligand 04 and Ligand 07 (G–L). Here, (A, D), (G, J) indicate protein ligands bind pocket; (B, E), (H, K) indicate hydrogen bonding; (C, F), (I, L) indicate 2D picture of active sites, respectively.

The term “protein‐ligand interaction” refers to the binding or interaction that takes place between a molecule of targeted protein and a relatively macro molecule that is known as a receptor. In the fields of drug discovery and computational biology, it determines how proteins interact with ligands.^59^, ^60^ During a molecular docking, the protein and ligand molecules are often represented as three‐dimensional structures. This allows to understand more accurately predict the specified amino acid which form during molecular docking. Within the context of the protein's binding site, the docking investigates several different potential orientations and conformations for the ligand. This investigation helps to get an idea of how strongly drug interact with receptor and identified the active amino acid residue, such as hydrogen bond donor‐acceptor area, and protein‐ligand binding posture.^61^ The results of the docking experiment were used to illustrate the protein‐ligand interaction (Figure 3). The graphical representation on the left showed the protein ligands bind pocket that was formed during the drug‐protein complex formation. The hydrogen bonding donor and acceptor were depicted in the second figure. In this instance, the donor region was described by the sky‐blue colour, and the acceptor region was described by the red colour. The final is a 2D representation of active sites where various types of amino acids are depicted as active moieties with various linkages (Figure 3). These entirely visual statistics were created by Pymol and the 2020 and discovery studio.

### 3.5 ADMET data investigation

To ensure the safety and quality of drug‐like molecules, absorption, distribution, metabolism, excretion, and toxicity (ADMET) of novel pharmacological compounds were predicted. The pharmacokinetics properties of these selected medications were analysed using the pkCSM website. The water solubility ranges are below −5.0 fr most of the compounds, indicating moderately soluble in an aqueous medium.^62^ The membrane permeability [as characterized by the colon cancer cell line (Caco‐2)] was shown to be at its highest (1.258 × 10–^6^) in Ligand 01 which means ligand 01 can easily pass the membrane. Since the human intestinal tract absorbs more than 90% of all chemicals, it should have a high G.I. absorption capability.^63^ The VDss ranges in the distribution section is also reported better performance, ligands 01, 03, and 07 can cross the BBB, while the rest molecules are unable to do so. When it comes to metabolism, CYP450 1A2 inhibitors and CYP450 2C9 substrates have been employed, and it has been observed that no selected medicines can either inhibit or interact with these enzymes. The unit of measurement for the total clearance rate is ml/min/kg. None of the ligands can be excreted by renal OCT2 in this instance, with ligand 02 having a minimum rate of total clearance ‐0.319  mL/min/kg and ligand 01 having a maximum rate of clearance 0.885 mL/min/kg. Finally, two parameters—AMES, and skin sensitization—were used to predict the toxicity. Only ligand 02 and 04 in the AMES parameter may have a carcinogenic effect, while none of them are predicted to the skin sensitization. Therefore, caution should be taken before taking the ligand 02 and 04 (Table 4).

**TABLE 4:** ADME and toxicity prediction of the selected ligands.

S/N	Absorption			Distribution		Metabolism		Excretion		Toxicity	
	Water solubility Log S	Caco‐2 permeability × 10^−6^	Human intestinal absorption (%)	VDss (human)	BBB permeability	CYP450 1A2 Inhibitor	CYP450 2C9 substrate	Total clearance (mL/min/kg)	Renal OCT2 substrate	AMES toxicity	Skin sensitization
01	−3.403	1.258	98	0.673	Yes	No	No	0.885	No	No	No
02	−2.607	−0.173	77	−0.428	No	No	No	−0.31	No	Yes	No
03	−4.726	0.982	94	0.535	Yes	No	No	0.686	No	No	Yes
04	−5.499	1.022	96	0.286	No	No	No	0.673	No	Yes	No
05	−4.372	0.962	94	−0.76	No	No	No	0.685	No	No	No
06	−4.888	1.181	97	0.412	No	No	No	0.764	No	No	No
07	−5.34	1.186	97	0.5	Yes	No	No	0.790	No	No	No
08	−5.14	0.948	96	0.164	No	No	No	0.244	No	No	No

### 3.6 Molecular dynamics simulation

**FIGURE 4:**
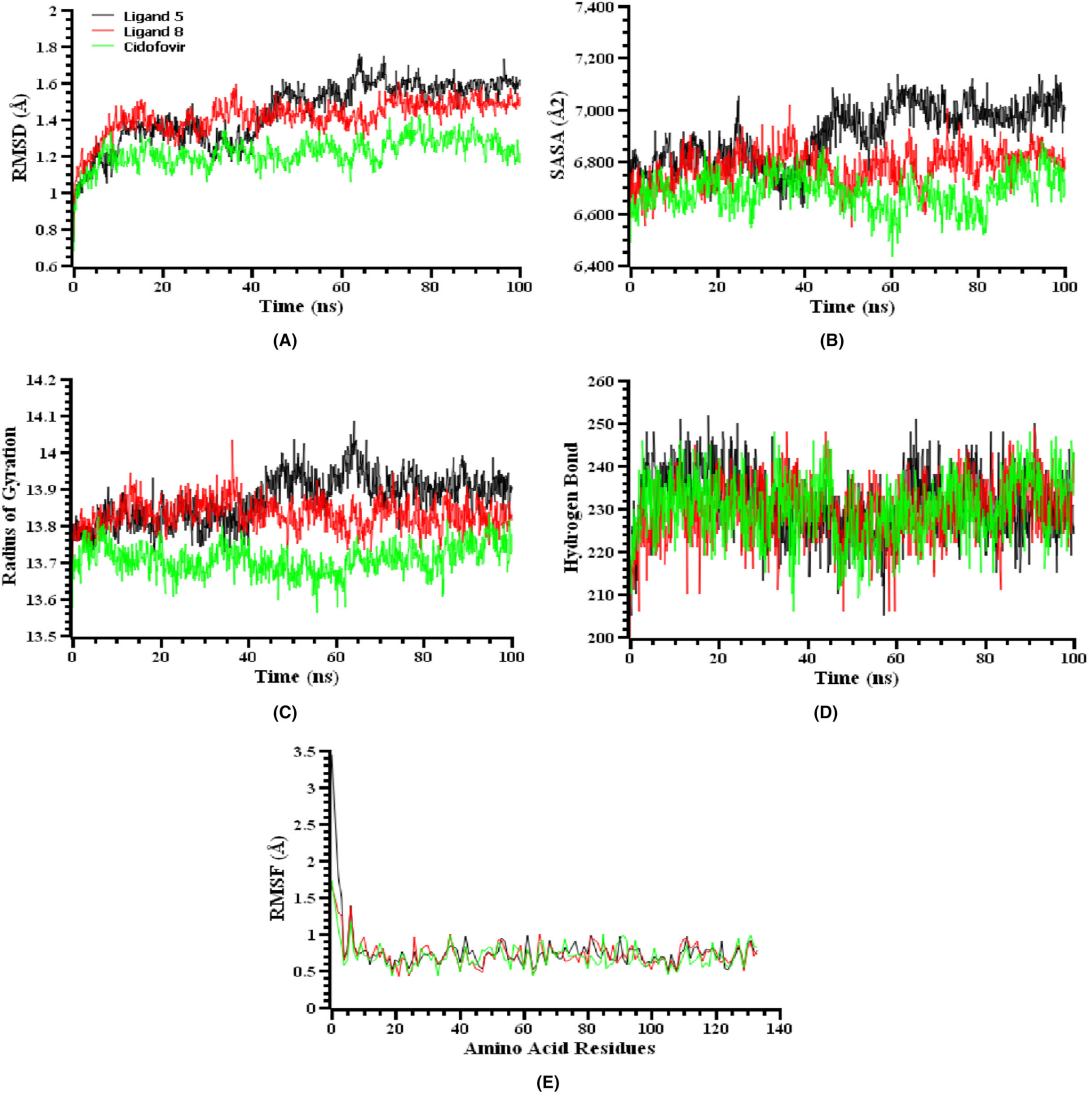
Molecular dynamics simulation of ligand 5, ligand 8 and standard against the selected protein of Monkeypox virus. Here, (A) Root mean square deviation, (B) solvent accessible surface area, (C) radius of gyration, (D) number of hydrogen bonds, and (E) root mean square fluctuation.

**FIGURE 5:**
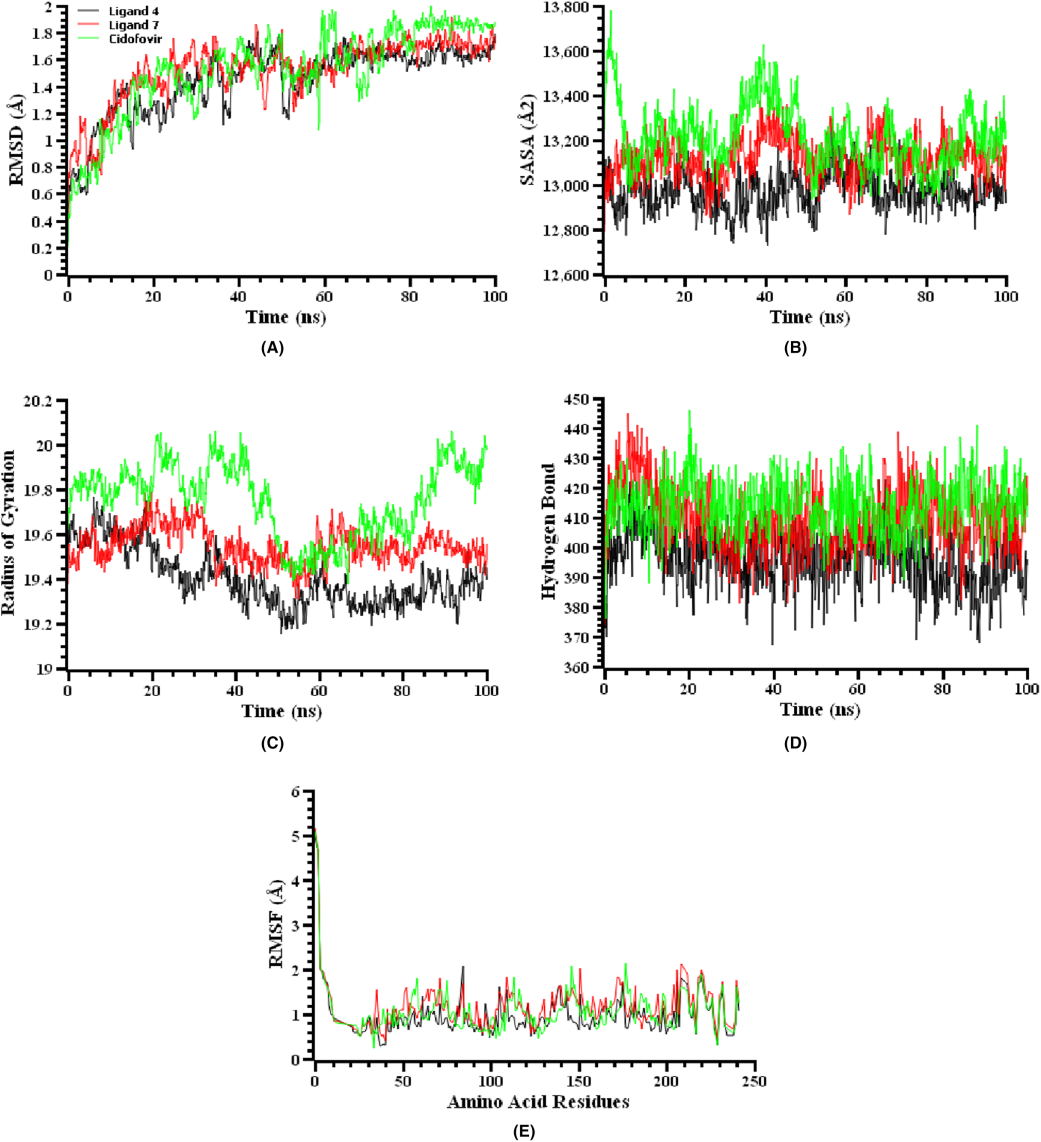
Molecular dynamics simulation of ligand 4, ligand 7 and standard against the selected protein of Marburg virus. Here, (A) Root mean square deviation, (B) solvent accessible surface area, (C) radius of gyration, (D) number of hydrogen bonds, and (E) root mean square fluctuation.

Two ligand‐protein complexes and the standard drug‐protein complex for both the mpox (PDB ID 4QWO) and Marburg (PDB ID 4OR8) virus were examined using molecular dynamics simulations to investigate their structural firmness and corroborate docking scenarios. The root‐mean‐square deviations (RMSDs) of the complexes containing ligand 5, ligand 8 and cidofovir (standard drug) for mpox (Figure 4A), and ligand 4, ligand 7, and cidofovir (standard drug) for Marburg virus (Figure 5A), exhibited an increase from their original locations. This rise can be attributed mostly to the inherent instability of the complexes. For mpox virus, it has the highest mean RMSD value in the case of ligand‐5, while it has the lowest mean RMSD value when standard‐cidofovir is bound. [Figure 4A]. For Marburg virus, it has a higher average RMSD value than the other two complexes when standard‐cidofovir is bound, while it has a lower average RMSD value when ligand‐7 is bound. [Figure 5A] Upon examining Figure 5A, it is evident that the RMSD value of the ligand 5 complex for the mpox virus experienced a significant increase during the first 10 ns of the simulation. Subsequently, there were substantial oscillations in the RMSD value up to 70 ns, with a notable peak occurring at 65 ns, representing the highest recorded value. Nevertheless, the simulation achieved equilibrium over the final 30 ns. Upon binding ligand‐8, the RMSD value displayed analogous behaviour to that of ligand‐5. However, in comparison to the other two ligands, the RMSD value exhibited an initial increase during the first 10 nanoseconds (ns) of the simulation. Subsequently, the average RMSD value decreased from this point until 80 ns and stabilized during the final 20 ns of the simulation. For Marburg virus, the ligand 4 exhibited a significant rise in RMSD value during the simulation, specifically within the first 20 ns. Between 20 ns and approximately 60 ns, the RMSD exhibited significant instability and changed irregularly. Nevertheless, it was noted that the structure achieved equilibrium within the final 40 nanoseconds of the simulation. Ligand 7 displayed comparable characteristics to ligand‐4, however it was less stable due to its higher average RMSD value. The standard‐cidofovir ligand displayed the highest degree of instability during the simulation in comparison to the other two ligands. Despite this, all mpox and Marburg virus complexes had RMSD values of less than 2.5 Å during the simulation, indicating that they remained stable but their stability differs from each other.

To ascertain how the surfaces of the mpox and Marburg virus complexes alter as a result of interactions with the ligand molecules, SASA values were calculated for each complex. The increasing value of SASA increases the surface area of the protein, but decreasing SASA causes protein truncations.^64^ It was evident from the higher SASA value than the other two complexes during the simulation period that the ligand 5‐4QWO complex for the mpox virus [Figure 4B] and the cidofovir‐4OR8 complex for the Marburg virus [Figure 5B] had a larger surface area. The cidofovir‐4QWO complex and the ligand 4‐4OR8 complex had lower SASA values on average in comparison to the other complexes for the mpox and Marburg virus, respectively. Ligand 5‐4QWO, ligand 8‐4QWO and cidofovir‐4QWO complexes for the mpox virus reached a steady state after 60 ns, 70 ns, and 80 ns, respectively, and maintained their stability for the remaining simulation time. Moreover, in the case of the Marburg virus, the ligand 4‐4OR8, ligand 7‐4OR8, and cidofovir‐4OR8 complexes reached a steady state after 70 ns, 75 ns, and 90 ns of simulation time, respectively, and remained stable for the remaining simulation time with only negligible fluctuation.

By employing the Rg values, it is possible to evaluate the degree of compactness or instability of protein complexes. A protein‐ligand complex with a lower radius of gyration (Rg) will exhibit greater stability, while a protein‐ligand complex with a larger Rg will be more prone to instability. The Rg value of the ligand 8‐4QWO was comparatively less fluctuating than the other two complexes for the mpox virus and showed a lesser Rg value on average than the ligand 5‐4QWO complex during the simulation period, indicating the higher stability of that complex [Figure 4C]. Besides, the cidofovir‐4QWO complex had the lowest Rg value on average than the other two complexes. However, in the case of the Marburg virus, the Rg values of cidofovir‐4OR8 were notably higher compared to the other two complexes. The simulation results showed consistently high Rg values, particularly between 20 and 40 ns, as well as in the last 10 ns of the simulation. These findings suggest that the structure of cidofovir‐4OR8 is highly unstable, as depicted in Figure 5C. The Rg values of ligand 4‐4OR8 and ligand 7‐4OR8 are comparatively lower, suggesting that they exhibit greater stability during the simulation period.

The stability and integrity of proteins depend on hydrogen bonds. Therefore, the docked complexes were carefully examined to assess the formation of hydrogen bonds. The complex formed by ligand 5‐4QWO, ligand 8‐4QWO, and cidofovir‐4QWO with mpox virus is depicted in Figure 4D. The Marburg virus is complexed with ligand 4‐4OR8, ligand 7‐4OR8, and cidofovir‐4OR8 in Figure 5D. A substantial number of hydrogen bonds were established, which maintained the stability of those complexes throughout the simulation duration.

Upon examining Figure 4D, it is evident that hydrogen bond creation is consistently strong for all three ligands throughout the initial 30 nanoseconds of the simulation. Notably, ligand 5 exhibited the highest level of hydrogen bond formation. Subsequently, while a decline in hydrogen bond production was noted during the middle phase of the simulation, the creation of hydrogen bonds for all three ligands experienced an increase in the final 20 ns of the simulation. This statement also applies to Figure 5D. Nevertheless, a greater occurrence of hydrogen bond formation was noted in the Marburg virus in comparison to the monkey pox virus.

**FIGURE 6:**
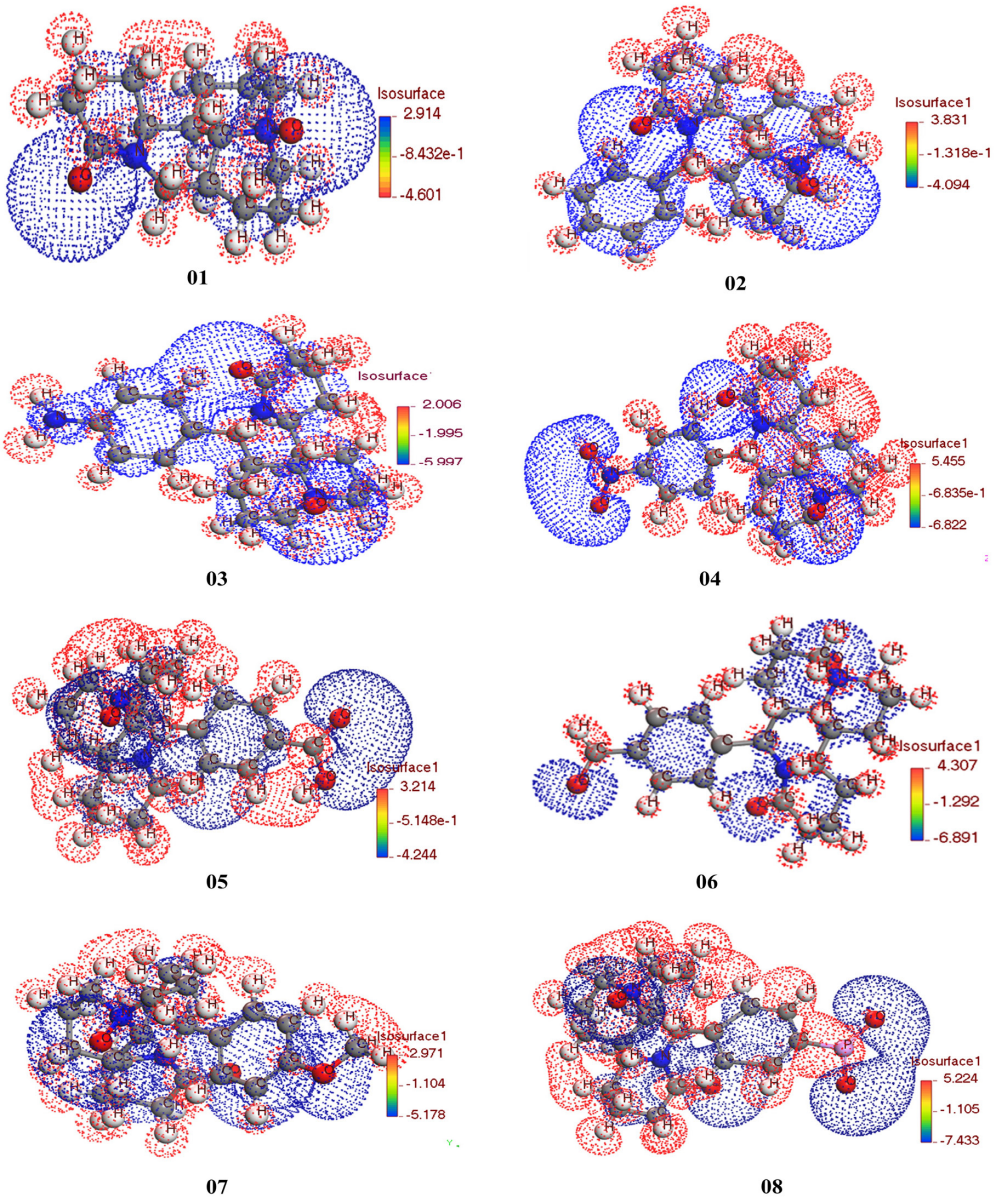
Electrostatic potential charge distribution map.

A further analysis was conducted to determine the proteins' flexibility across amino acid residues using RMSF values of the protein‐ligand complexes for both the mpox and Marburg viruses. The mpox and Marburg virus protein‐ligand complexes all had RMSF profiles lower than 2.5 Å, except for the first few amino acid residues [Figures 4E and 5E]. The RMSF plots of ligand‐protein interaction for each ligand, shown in Figures 5E and 6E, indicate that the C‐terminal residues exhibit a significantly high RMSF value. This is owing to the fact that these residues correspond to the tails or ends of the protein structure, which are characterized by high reactivity and freedom of movement. As all the complexes for both viruses had lower RMSF values during the simulation time, it can be concluded that they had a lower flexibility level, since lower RMSF values designate a higher stability of the complexes.^65^

### 3.7 Binding free energy calculation

After completing the binding energy calculations, it was observed that the obtained scores were superior compared to the standard. So, the binding free energy calculations were conducted for a better understanding.

For the mpox virus, the average binding free energy values for Cidofovir (standard), ligand 5, and ligand 8 were −31.40, −49.38 and −46.15 kJ/mol, respectively (Table 5). Regarding the Marburg virus, the average binding free energy values for Cidofovir (standard), ligand 4, and ligand 7 were −33.92, −45.62 and −41.21 kJ/mol, respectively (Table 5). Notably, the plausible ligand molecules exhibited higher average binding free energy than the standard complex for both viruses. This strongly suggests that the newly developed molecules possess greater stability in comparison to the standard molecules.

**TABLE 5:** MM‐PBSA binding free energy calculation of the top two screened molecules with the standard complex.

Target receptor	Complex	Average binding free energy (kJ/mol)
Monkeypox virus (PDB ID 4QWO)	05	−49.38
	08	−46.15
	Standard (Cidofovir)	−31.40
Marburg virus (PDB 4OR8)	04	−45.62
	07	−41.21
	Standard (Cidofovir)	−33.92

### 3.8 Frontier molecular orbitals and reactivity descriptor analysis

Frontier molecular orbitals and reactivity play an essential in understanding chemical molecules and active compounds. They provide valuable information about the energy gap, which indicates the strength of their chemical reactivity and their effectiveness in participating in chemical reactions.^66^, ^67^ As the hardness value increases, the molecule's susceptibility to degradation decreases, making it less suitable as an active medication. Conversely, biological activity is closely associated with the softness index and handedness, which are quantitatively opposite to each other.

According to Table 6, the energy gap for the primary compound (oxymatrine) is 8.289 eV. With the addition of side chains or functional groups, the energy gap decreases, resulting in values of 8.399, 7.542, 6.591, 7.393, 7.383, 8.128 and 6.572 eV for compounds 02, 03, 04, 05, 06, 07 and 08, respectively. Initially, the energy gap is 8.289 eV and decreases with the addition of functional groups. A higher energy gap between the HOMO‐LUMO indicates high stability and low reactivity. This is because the electron requires more energy to excite an electron from the HOMO to the LUMO, making it less likely to participate in chemical reactions.^68^ Compound 04 exhibits the smallest energy gap of approximately 6.591 eV, indicating that this derivative is chemically more unstable but more reactive than the others compound. At the same time, chemical compound 02 is showing the maximum level of energy gap (8.399 eV) which means this molecule are more stable but less reactive.

**TABLE 6:** HOMO, LUMO and chemical reactivity descriptors calculation.

No	I ≈ −ε_HOMO_	A ≈ −ε_LUMO_	Energy GAP = ε_HOMO_ − ε_LUMO_	Hardness (*ɳ*) ≈ ½ (ε_LUMO_ − ε_HOMO_) ≈ ½ (I − A))	Softness (*σ*) = 1/η
01	−8.432	−0.143	8.289	4.1445	0.2413
02	−8.379	−0.020	8.399	4.1795	0.2393
03	−7.614	−0.072	7.542	3.771	0.2652
04	−8.753	−2.162	6.591	3.2955	0.3034
05	−8.819	−1.426	7.393	3.6965	0.2705
06	−8.579	−1.196	7.383	3.6915	0.2709
07	−8.325	−0.197	8.128	4.064	0.2461
08	−8.781	−2.209	6.572	3.286	0.3043

In terms of compound bioactivity, lower softness values are more effective, while higher hardness values indicate greater stability. The addition of functional groups increases the softness value and decreases the hardness value. Compound 02 has the highest hardness value of 4.1795, while compound 08 has the lowest hardness value of 3.286. On the other hand, compound 08 has the highest softness value of 0.3043, while compound 02 has the lowest softness value of 0.2393. In summary, the description of the research highlights the importance of chemical identifiers and their relationship with energy gap, stability, and biological activity. The Frontier molecular orbital (HOMO and LUMO) diagram are given in Figure S1.

### 3.9 Electrostatic potential charge distribution map

The mapping of electrostatic potential (MEP) has emerged as an essential experiements for measuring and evaluating the reactivity of chemical species in a wide range of molecular interactions. This theoretical approach has proven effective in investigating and analysing physical, biological, and related processes, with a particular focus on the active site of orbitals.^69^ When exploring bioactive compounds, MEP provides valuable insights into the methodologies and active charge‐carrying regions of molecules. By doing so, it enhances our understanding of the attachment mechanisms between ligands and proteins. Figure 6, it becomes evident that the entire molecule contains an overall distribution of negative and positive charges, which can be divided into two distinct parts. At the connection points, the charges are measured at −8.432e‐1, −1.318e‐1, −1.995, −6.835e‐1, −5.148e‐1, −1.292, −1.104 and −1.105. In terms of colour representation, the negative charges are depicted in red, while the positive charges are indicated by blue. Regarding charge distribution, most of the inner region, including the ring structure and the molecule shown in Figure 6, exhibits a predominant blue colour. However, the outer part, which encompasses the hydrogen atoms, is characterized by a red colour.

The MEP analysis has proven to be a valuable method in understanding molecular reactivity, as it allows researchers to examine the charge distribution and active sites within a molecule.

## 4 CONCLUSION

This *in silico* study investigated to identify potential drugs for suppressing the mpox and Marburg virus pathogens through the computational approaches including molecular, docking, dynamics simulation, drug‐likeness assessment, DFT calculation, and ADMET profiling, among others. These analyses aimed to evaluate the suitability of the selected molecules as potential drugs. In the subsequent molecular docking experiments, the mpox and Marburg virus demonstrated the greatest binding energy. These binding energies indicate the strength of interaction between the proposed molecules and the target viral proteins. Considering the pharmacokinetics profile, the proposed molecules showed high absorption in the gastrointestinal tract. However, only a limited number of the compounds were predicted to penetrate the blood–brain barrier, while the majority were unable to do so. Except for Ligand 02 and 04, which exhibited AMES toxicity, and most of the molecules had a greater distribution rate and were unaffected by AMES toxicity. Based on the promising results obtained from this computational study, further investigation could be significant in wet lab.

## AUTHOR CONTRIBUTIONS

**Md. Rezaul Islam:** Conceptualization (equal); formal analysis (equal); investigation (equal); methodology (equal); project administration (equal); validation (equal); writing – original draft (lead). **Suvro Biswas:** Conceptualization (equal); investigation (equal); methodology (lead); project administration (equal); resources (equal); software (equal); writing – original draft (equal). **Ummy Amena:** Conceptualization (equal); data curation (equal); formal analysis (equal); methodology (equal); resources (equal); writing – original draft (equal). **Miadur Rahman:** Conceptualization (equal); data curation (equal); investigation (equal); methodology (equal); validation (equal); writing – original draft (equal); writing – review and editing (equal). **Shirmin Islam:** Investigation (equal); methodology (equal); project administration (equal); software (equal); validation (equal); writing – original draft (equal). **Md. Ariful Islam:** Data curation (equal); investigation (equal); methodology (equal); project administration (equal); software (equal); validation (equal); writing – review and editing (equal). **Md. Abu Saleh:** Conceptualization (lead); resources (equal); software (equal); supervision (equal). **Hesham M. Hassan:** Formal analysis (equal); validation (equal); writing – review and editing (equal). **Ahmed Al‐Emam:** Methodology (equal); writing – original draft (equal); writing – review and editing (equal). **Magdi E. A. Zaki:** Supervision (equal); writing – review and editing (equal).

## CONFLICT OF INTEREST STATEMENT

The authors declare no conflict of interest.

## Supporting information


Figure S1.

## Data Availability

All data generated or analyzed during this study are included in this article.
