# Supplementary figures and images for: Modified oxymatrine as novel therapeutic inhibitors against Monkeypox and Marburg virus through computational drug design approaches
Source: J Cell Mol Med. 2024 Sep 28;28(18):e70116. doi: 10.1111/jcmm.70116 (PMC11437895; doi:10.1111/jcmm.70116)

**Supplementary Figure S1:** Frontier molecular orbital: HOMO and LUMO


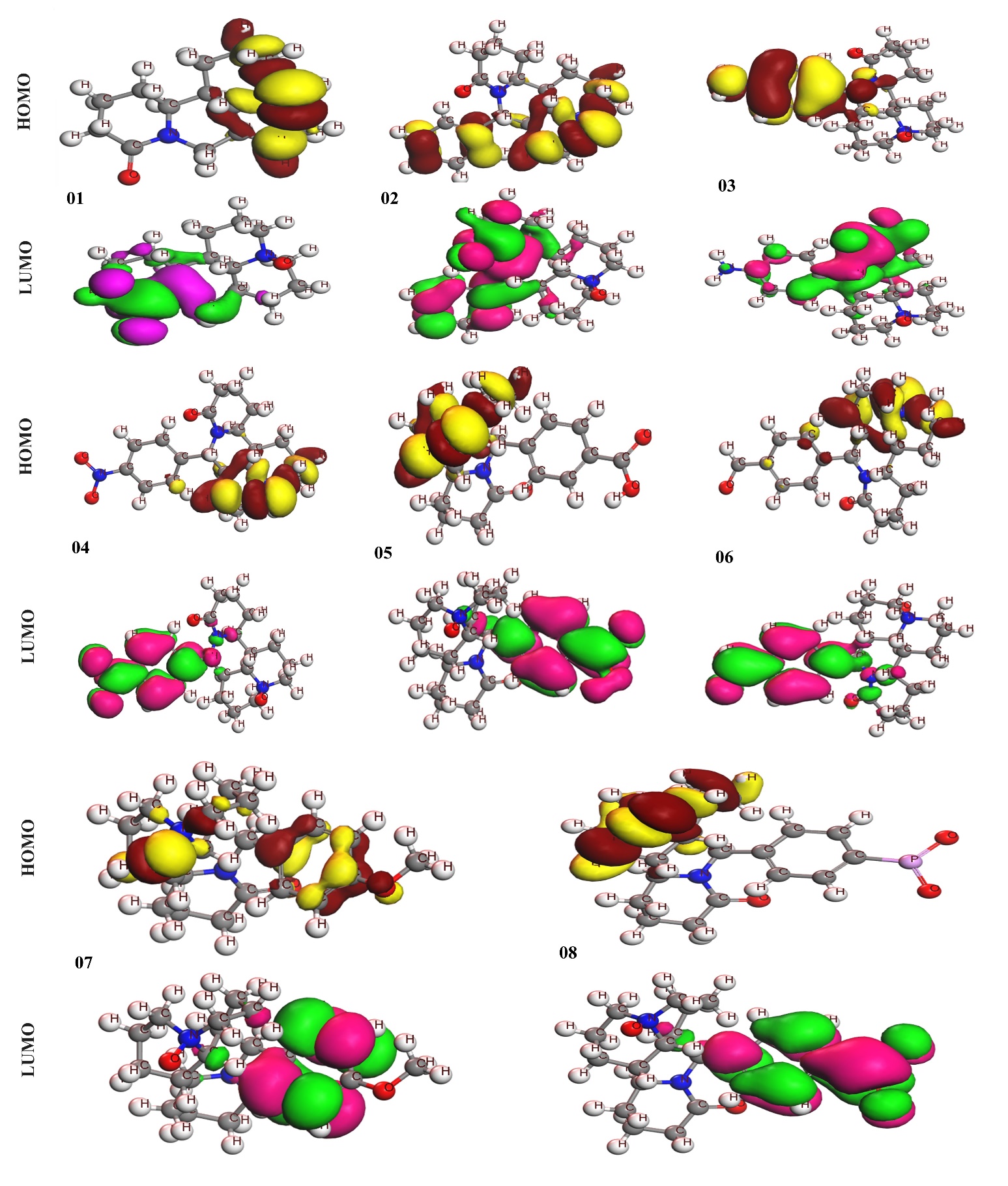

Supplement: Supplementary file 1 — Figure S1. [file JCMM-28-e70116-s001.docx]
